# Endophytic and Epiphytic Microorganisms as Biocontrol Agents: Mechanisms, Applications, and Metagenomic Approaches in Tomato Cultivation

**DOI:** 10.3390/molecules30183816

**Published:** 2025-09-19

**Authors:** Phathutshedzo Rakhalaru, Beverly Mmakatane Mampholo, Tshifhiwa Paris Mamphogoro, Mapitsi Silvester Thantsha

**Affiliations:** 1 Gastro-Intestinal Microbiology and Biotechnology Unit, Agricultural Research Council-Animal Production, Private Bag X02, Irene, Pretoria 0062, South Africa; rakhalarup@arc.agric.za; 2 Department of Biochemistry, Genetics and Microbiology, University of Pretoria, Private Bag X20, Hatfield, Pretoria 0028, South Africa; 3 Leguminous, Leafy and Fruit Vegetables Division, Agricultural Research Council-Vegetable, Industrial and Medicinal Plants, Private Bag X293, Roodeplaat, Pretoria 0001, South Africa; mampholom@arc.agric.za

**Keywords:** endophytes, epiphytes, biological control agents, *Solanum lycopersicum*, metagenomics

## Abstract

Tomato (*Solanum lycopersicum*) is an essential crop worldwide, yet it remains highly vulnerable to severe fungal and bacterial diseases. Traditional chemical-based disease management strategies, aimed at controlling these diseases face increasing scrutiny, due to concerns regarding pathogen resistance, environmental degradation, and potential health risks to humans. This has catalyzed the exploration of sustainable alternatives, with biological control emerging as a viable and promising strategy. Endophytic and epiphytic microorganisms are pivotal as biocontrol agents (BCAs), employing diverse strategies, such as generating antimicrobial substances, enzymes, and volatile organic compounds (VOCs), to suppress pathogen growth and enhance plant health. The efficacy of these antagonistic microorganisms is influenced by the cultivation systems employed, with significant variations observed between soil and hydroponic environments. Factors such as nutrient dynamics and microbial interactions play crucial roles in determining the success of BCAs in these different settings. The advent of metagenomic tools has transformed the landscape of microbial community research, facilitating the identification of functional genes associated with antagonistic activities and the adaptation of these microorganisms to diverse environmental conditions. This review aims to elucidate the potential of endophytic and epiphytic microorganisms in biological control, examining their mechanisms of action, the impact of cultivation systems on their effectiveness, and the application of metagenomics to optimize their use in sustainable disease management strategies for tomato crops.

## 1. Introduction

Tomato (*Solanum lycopersicum* L.) is a staple crop with high economic and nutritional value worldwide. However, the fresh market of this crop is vulnerable to numerous pathogens, including fungi and bacteria, that cause significant losses in crop yields during field cultivation and post-harvest storage [1,2,3]. The tomato crops are frequently affected by diseases caused by pathogens such as *Phytophthora infestans* (causing blight), *Fusarium oxysporum* (leading to wilt), *Alternaria alternata* (responsible for leaf spot), and *Pseudomonas syringae* pv. *tomato* (causing bacterial spot), which are responsible for severe yield reductions and compromise fruit quality, challenging sustainable agricultural practices [4,5,6]. Chemical treatments like pesticides and fungicides are widely used to eliminate and manage these pathogens; however, their excessive use can lead to chemical resistance, environmental degradation, and health risks for consumers [7,8]. Consequently, there is increasing interest in eco-friendly solutions, with a particular focus on biological control (biocontrol) methods for managing plant diseases.

Biocontrol utilizes beneficial microorganisms naturally present in the environment to suppress harmful pathogens, thereby reducing disease incidence and enhancing overall crop health [9]. Tomato crops host a variety of endophytic and epiphytic microorganisms, which have shown great potential as natural BCAs. Endophytic microorganisms like *Epicoccum nigrum* SGT8.6, *Bacillus amyloliquefaciens*, and *Trichoderma harzianum* enhance plant growth and disease resistance. Epiphytic microbes such as *Aureobasidium pullulans* SUG4.1, *Beauveria bassiana*, and *Cladosporium herbarum* contribute to plant defense and microbial balance [10,11,12]. The composition of these microbial communities varies with environmental conditions and tomato varieties. These microorganisms may exhibit antagonistic activity against plant pathogens, often through the synthesis of bioactive metabolites and enzymes that inhibit pathogen growth [13]. Additionally, they may produce VOCs that enhance biocontrol effects and suppress pathogen development [14]. The success of these biocontrol agents relies on various factors, including environmental conditions, microbial diversity, and cultivation systems [15,16]. In soil-based cultivation systems, microbial interactions and nutrient availability differ significantly from hydroponic systems, which rely on water-based nutrient solutions. These differences in cultivation systems can impact the survival, activity, and biocontrol effectiveness of the microorganisms, as well as their interactions with both pathogens and plants [12,17]. Numerous biological control agents are currently available; however, additional research is necessary to identify potential microorganisms and their natural products that exhibit broad-spectrum antagonistic activity for effective crop disease management.

To advance biocontrol strategies, metagenomic approaches have become valuable for investigating microbial genes that contribute to antagonistic activity. Through metagenomics, researchers can explore diverse microbial communities, identifying genes responsible for the production of compounds that inhibit pathogens and improve biocontrol effectiveness [18]. By analyzing the genetic potential of endophytic and epiphytic microorganisms, metagenomics provides insights into microbial diversity, functionality, and adaptability across different cultivation environments [19,20]. This genomic knowledge is essential for creating biocontrol strategies tailored to specific pathogens and agricultural conditions, supporting sustainable disease management in tomato cultivation.

This review aims to explore the potential of endophytic and epiphytic microorganisms as BCAs against fungal and bacterial pathogens affecting tomatoes. It focuses on their mechanisms of action, the influence of cultivation systems on their efficacy, application and commercialization, as well as metagenomic approach to enhance understanding of microbial diversity and functionality for developing sustainable disease management strategies.

## 2. Pathogens Affecting Tomatoes

**Figure 1:**
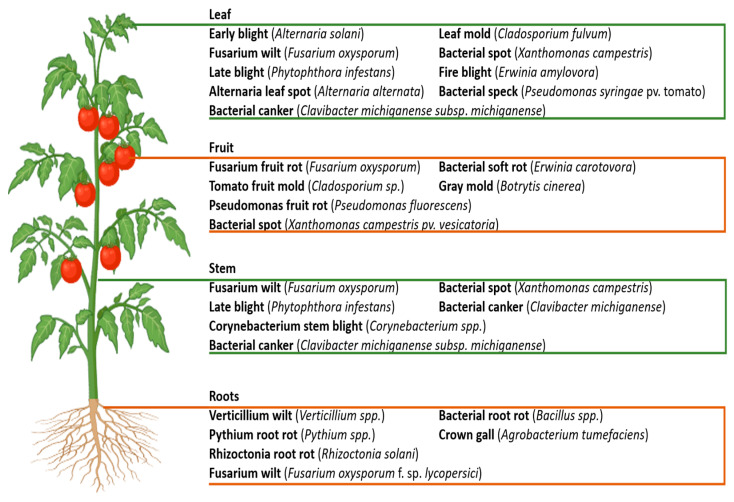
Overview of fungal and bacterial diseases affecting tomato plants: Illustration of major fungal and bacterial diseases impacting tomato leaves, fruits, stems, and roots. Leaf diseases include fire blight, late blight, leaf mold, bacterial spot, and Fusarium wilt. Fruit diseases encompass anthracnose, gray mold, bacterial soft rot, and bacterial spot. Stem diseases include bacterial soft rot, Fusarium fruit rot, and gray mold. Root diseases feature Fusarium wilt, Verticillium wilt, Pythium root rot, and crown gall, illustrating affected plant parts.

Pathogens from fungi and bacteria pose significant threats to tomato crops, significantly affecting yield and quality. These pathogens lead to various destructive diseases that complicate sustainable cultivation practices. This section reviews the major fungal pathogens affecting these crops, focusing on the diseases they cause and their impacts on crop productivity (Figure 1).

### 2.1. Major Fungal Pathogens

One of the most damaging fungal pathogens affecting tomatoes is *Alternaria solani*, the cause of early blight, which leads to leaf drop and reduced fruit yield [21]. Another serious threat is *F. oxysporum*, responsible for Fusarium wilt, which results in vascular wilting. One of the clinical signs of a disease in young plants is the wilting of lower leaves, accompanied by a loss of pigmentation, which ultimately results in death of the plant [22]. *P. infestans* is responsible for late blight disease, posing a significant threat to tomatoes, particularly in humid environments. This pathogen can rapidly spread and devastate crops, leading to severe yield losses. Late blight is mostly known to have caused major crop failures in the 19th century, highlighting its potential for widespread damage [23,24]. Additionally, *Cladosporium fulvum*, the causal agent of leaf mold, primarily affects the leaves of tomato plants, leading to yellowing, chlorosis, and defoliation. *C. fulvum* also reduces photosynthesis and affects the overall plant health [25]. Lastly, *Botrytis cinerea*, the causal agent of gray mold, infects ripe tomatoes, with symptoms that are bruised or damaged. This fungus often leads to significant damage during storage and transport, reducing the shelf life and marketability of tomatoes [26]. *Rhizoctonia solani* is a significant soil-borne pathogen that causes cotton root rot in solanaceous crops, particularly under stressed conditions, leading to poor root development and eventual plant death. The pathogen’s ability to cause significant damage has been documented in various countries, including Turkey and Mexico, where it has been linked to substantial crop losses [27,28].

### 2.2. Major Bacterial Pathogens

Bacterial pathogens significantly impact the health and yield of tomato crops, with several species being particularly notorious for causing diseases. Among these, *Xanthomonas campestris* pv. *Vesicatoria* (Xcv) is a prominent pathogen responsible for bacterial spots in solanaceous crops. It is known for causing small water-soaked lesions on leaves, stems, and fruit. This pathogen has been shown to possess various virulence factors, including type II secretion systems, which are crucial for its pathogenicity [29].

Another significant group of bacterial pathogens belongs to the *Pseudomonas* genus, which includes various species capable of causing diseases in crops such as tomatoes. For instance, *P. syringae* pv. and *Pseudomonas cichorii* also affect multiple solanaceous plant species, causing leaf spots and blights. These pathogens typically initiate infection through water-soaked lesions on leaves, which eventually turn brown and necrotic, leading to defoliation under severe conditions [30,31]. The interactions between *Pseudomonas* and plant hosts can lead to beneficial and detrimental outcomes, depending on the specific strains and environmental conditions. Beneficial strains, such as *Pseudomonas fluorescens*, promote plant growth by producing siderophores that enhance iron availability, as well as antimicrobial compounds that suppress soil-borne pathogens [32]. On the other hand, pathogenic strains like *P. syringae* can cause significant damage to crops by producing toxins such as coronatine, which facilitates infection by suppressing crop immune responses [33].

*Clavibacter michiganensis* subsp. *michiganensis* (Cmm) is a major bacterial pathogen of tomatoes, causing bacterial canker, a disease that leads to substantial economic losses. This pathogen is particularly challenging to manage because it can infect plants systemically and often shows resistance to various control methods [34,35]. *Ralstonia solanacearum* is responsible for bacterial wilt in various crops. This bacterium can survive in the soil. Infection by *R. solanacearum* occurs through the roots, causing rapid wilting and plant death. The *R. solanacearum* bacterium produces exopolysaccharides and other virulence factors that facilitate its invasion and colonization of host tissues [36]. Lastly, *Erwinia carotovora*, known for causing soft rot, affects a variety of vegetables, including tomatoes. This bacterium thrives in moist conditions and can rapidly degrade plant tissues, leading to significant post-harvest losses [37].

## 3. Microbial Biological Control in Sustainable Agriculture

Microbial biological control serves as a foundation of sustainable agriculture, utilizing beneficial microorganisms to control pathogens in crops. These beneficial microorganisms (bacteria and fungi) act as natural antagonists to harmful pathogens, decreasing the dependence on chemical pesticides [38]. While microbial biocontrol is already widely applied, research is ongoing to optimize its use for maximum efficacy in different crop systems. Biocontrol agents also support environmentally friendly farming practices by enhancing plant health and protecting crops, contributing to ecological balance, promoting soil health, and improving food safety by minimizing chemical residues on agricultural products [39]. The integration of microbial biocontrol into farming systems remains essential for advancing sustainable and resilient agricultural practices.

### Advantages and Challenges of Microbial Biocontrol over Chemical Treatments

Microbial biocontrol has advantages that make it a promising alternative to chemical treatments. These agents provide a range of benefits that chemical treatments cannot match, particularly in terms of environmental impact, specificity, and long-term soil health. Microbial biocontrol agents typically have a lower toxicity profile than synthetic pesticides and fungicides, which can lead to less harm to non-target organisms [40]. For instance, the use of microbial agents can enhance soil microbial diversity, which is crucial for maintaining soil health and fertility [41]. In contrast, chemical fungicides often disrupt natural microbial communities, reducing beneficial microbes that suppress pathogens [42]. This disruption can weaken natural disease suppression in the soil, leading farmers to apply more chemical inputs to compensate, thereby creating a dependency cycle that undermines the principles of sustainable agriculture.

Additionally, biocontrol agents have been reported to promote plant growth and enhance tomato root development by stimulating morphological changes such as increased root branching, root length, greater root hair formation, and thicker root systems [43]. These changes improve nutrient uptake and plant vigor. Furthermore, biocontrol agents such as *Bacillus* and *Trichoderma* species produce bioactive compounds that induce systemic resistance, enabling tomato plants to defend themselves more effectively against future pest and pathogen attacks [44,45]. For example, certain bacteria and fungi can produce antibiotics or other inhibitory compounds that confer resistance to specifically targeted pathogens, thereby reducing disease incidence [46,47]. Saylan [48] reported that the fungicide captan inhibits germination and root growth in solanaceous crops, suggesting that such chemicals can disrupt critical enzymatic processes necessary for crop growth. Moreover, biocontrol methods are associated with a lower risk of pathogen resistance development compared to chemical treatments. The diverse mechanisms employed by microbial biocontrol agents, such as competition, parasitism, and the production of antimicrobial compounds, make it challenging for pathogens to develop resistance [49,50].

The use of BCAs significantly reduces farm workers’ exposure to hazardous chemicals, which is an important public health concern. Exposure to synthetic pesticides has been strongly associated with acute poisoning, respiratory and neurological issues, and long-term chronic diseases among agricultural workers [51]. A systematic review by Muñoz-Quezada [52] confirmed that chronic occupational exposure to organophosphates impairs neuropsychological functioning, including memory, attention, executive function, psychomotor speed, and visuospatial abilities, underscoring the risks of continued reliance on chemical pesticides. In contrast, the adoption of microbial biocontrol methods reduces these health risks by limiting pesticide use and the overall chemical load in the working environment. Importantly, a 3-year follow-up study of 579 greenhouse workers exposed to microbial control agents (*B. thuringiensis*, *V. lecanii*, and *T. harzianum*) found no association between exposure and respiratory symptoms, lung function decline, or bronchial responsiveness, despite long-term occupational contact [53]. This study supports the relative safety of BCAs compared to synthetic chemical fungicides and pesticides. The use of BCAs contributes to safer food consumption, reduced dietary intake of toxic compounds, and supports sustainable food production systems [54].

Even with all the above-mentioned advantages, the application of microbial biocontrol is not without challenges. One of the primary challenges is the efficacy and consistency of microbial BCAs. Their performance can be highly variable, influenced by environmental factors such as temperature, carbon and nitrogen sources, pH, humidity, and soil conditions [55,56]. Puopolo et al. [57] emphasized that temperature significantly affects the biocontrol efficacy of *Lysobacter capsici* AZ78 against *P. infestans* on tomato leaves. The strain effectively colonized leaves and controlled the pathogen at intermediate temperatures, while exposure to higher temperatures severely impaired its biocontrol performance. The study conducted by Wang et al. [58] showed that the efficacy of *Bacillus cereus* strain BCM2 is highly influenced by environmental factors such as soil type, water content, pH, and organic matter levels.

Another challenge is the specificity of many BCAs which often target specific pathogens. This makes them less versatile in crop production systems where multiple threats may occur at the same time. As a result, farmers may see BCAs as less practical or appealing compared to chemical alternatives [59]. Studies have shown that *P. fluorescens* can suppress several soil-borne fungal pathogens such as *Fusarium* spp. and *Rhizoctonia* spp., largely through the production of antifungal metabolites (e.g., phenazines, pyoluteorin) [60]. However, its effectiveness is limited against certain aggressive bacterial pathogens like *R. solanacearum*, as its predominant mode of action is antifungal rather than antibacterial. *B. pumilus* strain 3–19 has been reported to exhibit antimicrobial activity against the phytopathogenic bacteria *Pectobacterium atrosepticum* SCRI1043 and *Xanthomonas vesicatoria* DSM22252. However, no antagonistic activity has been reported against the phytopathogenic fungi *F. oxysporum* DR57 and *A. solani* 12RKL15 [61]. This highlights that the biocontrol potential of BCAs depends strongly on the specific strain and pathogen interaction.

Another significant challenge is the slower action of BCAs. In contrast to chemical pesticides, which can provide immediate results, microbial agents often require a longer period to establish and exert their effects on pests and pathogens [62]. This delay can be a significant drawback for farmers needing rapid control solutions. Microbial biocontrol agents typically operate through various mechanisms [63]. However, these processes often take time to manifest. For instance, when *Bacillus subtilis* is applied to the soil, it may take several days to establish itself in the rhizosphere and begin exerting its biocontrol effects against pathogens like *F. oxysporum* [64]. This delay can be particularly problematic when immediate disease control is necessary, such as during active disease outbreaks.

These challenges limit their practical and commercial application. Many strains exhibit strong antagonistic activity in vitro, yet their efficacy often diminishes in vivo due to environmental factors such as soil composition, temperature, humidity, and competition with native microbial communities. Maintaining strain stability and viability during seed coating, storage, and transport is also critical, as many microbes are sensitive to environmental stress. Regulatory approval and safety testing add additional complexity, requiring thorough evaluation to ensure environmental and human safety. Formulation and delivery methods whether seed coatings, soil drenches, or foliar applications must support microbial survival and functionality throughout the product’s shelf life [65].

Despite the challenges associated with BCAs, including slow action, specificity, environmental dependency, and inconsistent performance, their advantages often outweigh these limitations. The long-term sustainability of reduced environmental impact, reduced health risk, and reduced risk of resistance development make BCAs a promising alternative to chemical pesticides.

## 4. Endophytic and Epiphytic Microorganisms in Biocontrol

Tomatoes host various endophytic and epiphytic microorganisms that inhabit their internal and external tissues. Most research focuses on well-known epiphytic beneficial microbes that colonize the rhizosphere of crops like tomatoes. Recently, endophytes have gained attention for their potential as antagonists against pre- and post-harvest diseases in crops. Beyond their natural presence, BCAs can also be introduced before or during planting, allowing them to colonize the crop early and provide protection throughout plant development. By leveraging their natural ability to suppress pathogens and enhance crop health, endophytes and epiphytes are gaining recognition as sustainable alternatives to chemical treatment [66].

### 4.1. Endophytic Microorganisms in Biocontrol

Endophytic microorganisms are beneficial microbes that inhabit the internal tissues of crops without causing harm. They colonize diverse plant organs, including leaves, stems, roots, fruits, and reproductive structures such as flowers and seeds [66]. The diversity of endophytes often varies among organs, with roots typically showing the highest richness due to their interaction with the rhizosphere and root exudates that provide a nutrient-rich environment for microbial colonization [67].

Endophytes can enter plants through different pathways. Some are seed-borne, allowing them to establish early associations as the seed germinates and then spread into developing roots and shoots. Others originate from the rhizosphere, where they enter through root hairs, cracks, or wounds and may spread systemically via the vascular system to above-ground tissues [68]. Reproductive organs, including flowers and seeds, also act as reservoirs, supporting vertical transmission of endophytes across plant generations [69]. These unique characteristics make endophytes essential contributors to sustainable agricultural practices, offering promising alternatives to chemical inputs.

Endophytic microorganisms are beneficial microbes that live within the internal tissues of crops. They can colonize different crop tissues such as leaves, stems, roots, fruits, and even reproductive organs such as flowers and seeds, without harming the plant [66]. The diversity of endophytes varies across different plant organs, with the roots showing the highest diversity. Each organ may host distinct endophytic communities influenced by factors such as plant physiology, nutrient availability, and environmental conditions [70]. This variation is influenced by factors such as root exudates, which provide a rich source of nutrients for microbial colonization, and by the direct interaction of roots with the soil microbiome [71]. Endophytes are increasingly recognized as valuable agents in agricultural biocontrol because they suppress plant pathogens, promote plant growth, and enhance resistance to environmental stresses. These unique characteristics position them as essential components in sustainable farming strategies [72].

Endophytes are classified into two categories: obligate and facultative endophytes. Obligate endophytes depend entirely on their plant host for survival and cannot live outside the plant tissues. They form a permanent symbiotic relationship with the plant, providing it with various benefits [73]. On the other hand, facultative endophytes are more versatile, as they can survive both within the plant and in the external environment. Although they do not require the plant for survival, facultative endophytes can still confer advantages to the plant by suppressing pathogens [74].

Various endophytes of tomatoes have been found to work as effective biocontrol agents, suppressing a range of plant diseases. *Trichoderma* spp. are among the most extensively studied fungal endophytes due to their remarkable biocontrol properties. Species like *T. harzianum* are particularly effective in suppressing soil-borne pathogens such as *Fusarium* spp., *Verticillium* spp., *R. solani*, and *Pythium* spp., which are responsible for root rot, wilt, and other soil-related diseases in solanaceous crops [75,76,77]. A study by Sallam et al. [78] demonstrated that the obligate endophytes, *P. putida* and *Pseudomonas mediterranea*, isolated from healthy tomato crops, exhibited the most potent inhibitory effect against tomato early blight. In dual-culture tests, these strains effectively suppressed the growth of *Curvularia lunata*, *A. alternata*, and *A. solani*, highlighting their potential as biocontrol agents against a range of plant pathogens. An endophyte, *Paecilomyces formosus* ED, demonstrated significant activity in inhibiting the growth of a variety of pathogens, including *Fusarium verticillioides*, *R. solani*, *F. oxysporum*, *Rhizopus oryzae*, *Rhizopus stolonifer*, *B. cinerea*, and *Fusarium graminearum* [79]. These findings highlight that a single endophyte can effectively combat a variety of crop pathogens. This demonstrates the potential of endophytes as versatile BCAs that are capable of providing broad-spectrum protection to plants against multiple diseases. 

*B. pumilus* is a bacterium that has shown significant potential as a BCA in tomato plants, both as an endophyte and epiphyte, demonstrating its role as a facultative endophyte. One study reported the antagonistic activity of *B. pumilus* isolated from tomato leaf tissues against *P. syringae* pv. *tomato* NS4 [80]. This highlights its ability to suppress pathogens from within the plant as an endophyte. In a separate study, *B. pumilus* isolated from the surface of healthy tomato leaves exhibited antagonistic activity against *X. vesicatoria* [81]. This suggests that *B. pumilus* can also thrive on plant surfaces and offer protection against foliar diseases. These findings collectively demonstrate that *B. pumilus* is a versatile facultative endophyte that is capable of providing disease resistance both internally (as an endophyte) and externally (as an epiphyte), making it a strong BCA for integrated disease management in tomato cultivation. These examples of endophytic bacteria and fungi highlight the diverse capabilities of endophytes in providing natural and effective protection against plant pathogens, reinforcing their role as valuable tools in sustainable agricultural practices [80,81].

### 4.2. Epiphytic Microorganisms in Biocontrol

Epiphytic microorganisms, which colonize on the external surfaces of plants, play an important role in the biocontrol of plant diseases. These microorganisms include a diverse array of bacteria, fungi, and yeasts, and engage in complex interactions with both their plant hosts and the pathogens associated with them [82].

Several studies have highlighted the effectiveness of specific epiphytic microorganisms in controlling diseases in tomatoes. A study by Junior et al. [83] investigated four epiphytic microorganisms (*Aspergillus* sp., *Cellulomonas flavigena*, *Candida* sp., and *Cryptococcus* sp.) isolated from leaf and stem surfaces (phyllosphere) as potential biological control agents against *P. infestans*, the causative agent of tomato late blight. Their findings highlighted the ability of these microorganisms to antagonize the pathogen, presenting a promising alternative to chemical control for managing late blight in tomato cultivation. A study on epiphytic *B. bassiana* explored its effectiveness as a dual biocontrol agent against tomato pathogens, *B. cinerea* and *A. alternata*, and the aphid pest *Macrosiphum euphorbiae*. Among ten tested isolates, four demonstrated strong biocontrol efficacy of *B. bassiana*, with the Bb716 strain standing out for its dual functionality in controlling biotic stressors and promoting plant growth [84].

Another study assessed the biocontrol potential of epiphytic *Paenibacillus macerans* and *B. pumilus* against bacterial spot and early blight in tomato plants. The study findings showed that both bacteria reduced disease severity and decreased the population of phytopathogenic bacteria by 70% on the plant surface. Overall, epiphytic *P. macerans* and *B. pumilus* demonstrated strong biocontrol activity, suggesting their potential as effective agents for managing tomato diseases. A study by Panebianco et al. [85] reported that epiphytic bacterial strains isolated from the surfaces of tomato fruits exhibited antagonistic activity against several post-harvest pathogens. The predominant epiphytic genera identified by 16s rRNA were *Bacillus*, *Pseudomonas*, *Citrobacter*, and *Enterobacter*. These strains demonstrated inhibitory activity against *B. cinerea* (gray mold), *A. alternata* (Alternaria rot), *C. michiganensis* subsp., *michiganensis* (bacterial canker), *P. syringae* pv. *tomato* (bacterial speck), and *Xanthomonas euvesicatoria* pv. *perforans* (bacterial spot). The biocontrol potential of these epiphytic strains was confirmed through both in vitro and in vivo assays, showing their ability to suppress pathogen growth and reduce disease incidence on tomato fruits.

## 5. Metabolomics Approach in Tomato Biocontrol

Metabolomics provides insights into plant–microbe interactions by identifying biochemical changes in plants treated with BCAs [86]. Advanced analytical techniques such as Nuclear Magnetic Resonance (NMR) spectroscopy, Gas Chromatography-Mass Spectrometry (GC-MS), and Liquid Chromatography-Mass Spectrometry (LC-MS) allow for the detection and quantification of metabolites involved in plant defense and pathogen suppression [87,88].

Metabolomics-driven studies have enabled the identification of key metabolic pathways activated by BCAs in tomatoes. Metabolomics also plays a crucial role in evaluating the metabolic shifts occurring in plants upon pathogen attack and biocontrol treatment, thus helping to refine biocontrol strategies for enhanced disease resistance [89]. By applying metabolomics, researchers can characterize the metabolic profiles of tomato crops under biocontrol treatments, offering a deeper understanding of the mechanisms involved.

### 5.1. Biocontrol Mechanisms and Key Metabolites of Microbial Endophytes and Epiphytes

Microbial endophytes and epiphytes employ a variety of antagonistic mechanisms that can be broadly classified into both direct and indirect mechanisms. Direct mechanisms involve the immediate action of these microbes in suppressing or eliminating pathogens, through the production of antimicrobial compounds, enzymes, or competition for space and resources (Table 1). On the contrary, indirect mechanisms often involve the activation of plant defense systems, where microbial interactions stimulate the plant’s immune response, providing long-term protection against pathogens [9]. Together, these strategies form a comprehensive approach to disease management, demonstrating the valuable role of microbial endophytes and epiphytes in agricultural ecosystems. These strategies are essential for understanding how microbial communities help defend plants and improve crop protection. By directly targeting pathogens or enhancing plant immune responses, endophytes and epiphytes play a key role in disease management [72].

#### 5.1.1. Production of Antimicrobial Compounds

One of the key mechanisms by which microbial endophytes and epiphytes protect plants from pathogens is through the production of antimicrobial compounds (Table 1). For example, endophytic bacteria, particularly those from the genus *Bacillus*, have been extensively researched for their potential as biocontrol agents against various plant pathogens [90]. A prominent example is *Bacillus subtilis*, which is known to produce a variety of antimicrobial compounds, including lipopeptides. Lipopeptides are amphipathic molecules with both hydrophilic and hydrophobic properties [91]. These compounds are effective against pathogens such as *R. solani* [90]. They can disrupt the cell membranes of pathogens, leading to leakage of intracellular contents and cell death. In addition to direct antimicrobial activity, lipopeptides also induce plant immune responses, enhancing systemic resistance [91]. Examples of lipopeptides produced by microbial biocontrol agents, such as *Bacillus* spp., *Pseudomonas* spp., and *Streptomyces* spp., include Iturin, Surfactin, and Fengycin. These compounds play a vital role in controlling plant pathogens through their antimicrobial properties [92,93].

*P. fluorescens* produces phenazines, which are nitrogen-containing antimicrobial compounds with significant biocontrol potential. Key phenazines include phenazine-1-carboxylic acid, 2-hydroxyphenazine, and phenazine-1-carboxamide, which suppress tomato soil-borne pathogens such as *F. oxysporum*, *Pythium ultimum*, and *R. solani*. These compounds generate reactive oxygen species, disrupt pathogen electron transport, permeabilize cell membranes, and inhibit biofilm formation. In crop production, phenazine-producing *P. fluorescens* enhances plant health by reducing diseases, promoting root growth, and inducing systemic resistance in crops like tomatoes and potatoes [94,95,96].

Some endophytes and epiphytes used in biocontrol produce actinomycin D, a specialized antibiotic that works by binding to deoxyribonucleic acid (DNA) and blocking ribonucleic acid (RNA) synthesis [97,98]. This leads to stopping protein synthesis and ultimately, bacterial cell death. Due to its mechanism of action, actinomycin D is highly effective against a wide range of bacterial pathogens. Certain species of *Streptomyces* produce actinomycin D and have shown significant potential as antibacterial agents in the biocontrol of tomato pathogens [93]. A study by Ling et al. [99] demonstrated that actinomycin D, produced by the *Streptomyces* sp. NEAU-HV9, significantly reduced the impact of *R. solanacearum* on tomato seedlings.

Phenolic compounds are among the antimicrobial secondary metabolites produced by certain antagonistic microorganisms, including bacteria and fungi, to suppress crop pathogens (Table 1). They also play critical roles in tomato defense responses at different stages of growth. For instance, chlorogenic acid, caffeic acid, and flavonoids disrupt pathogen cell walls, compromise membrane integrity, and inhibit key metabolic processes, while also acting as antioxidants that reduce oxidative stress [100,101]. Salicylic acid, although primarily a plant-derived signaling molecule, can also be induced by microbial interactions and functions as a central regulator of systemic acquired resistance, priming defense-related gene expression and strengthening the plant’s ability to withstand future pathogen attacks [102]. Importantly, the ability to produce or induce these phenolic compounds is isolate-specific, for example, certain strains of *B. amyloliquefaciens* and *P. fluorescens* have been shown to elicit or contribute to the accumulation of SA and phenolic derivatives in tomato plants, enhancing resistance against fungal pathogens such as *Fusarium* spp. [103,104].

#### 5.1.2. Production of Volatile Organic Compounds

Volatile organic compounds play a critical role in inhibiting pathogen growth, inducing plant defenses, and promoting overall plant health. VOCs produced by microbial endophytes and epiphytes, including alcohols, ketones, aldehydes, and sulphur compounds, can directly suppress pathogenic microorganisms or enhance plant resistance through systemic induction of defense responses [105] (Table 1). VOCs are generally considered safe and non-toxic to humans when used within appropriate limits. For instance, 2,3-butanedione, commonly employed as a food additive, has been shown to pose no health hazards [106].

Studies have shown that both endophytic and epiphytic microorganisms associated with tomato plants produce diverse volatile organic compounds (VOCs) that contribute to their biocontrol activity. For example, epiphytic *B. subtilis* and *B. amyloliquefaciens* strains produce acetoin and 2,3-butanediol, which can induce systemic resistance (ISR) and enhance pathogen suppression [107,108,109]. *Lysobacter capsici* AZ78 emits mono- and dialkylated methoxypyrazines against *P. infestans* [110,111]. Endophytic fungi and bacteria, such as *Trichoderma harzianum*, *Pseudomonas gessardii*, and *Streptomyces* spp., also release VOCs including 6-pentyl-2H-pyran-2-one, variable phenazine derivatives, and 2-methylisoborneol, respectively. These VOCs contribute to ISR, mycoparasitism, and pathogen inhibition [10,95,112]. Additionally, dual epiphytic/endophytic strains like *P. fluorescens* produce acetophenone, dimethyl trisulfide, and phenazine derivatives, which, together with antimicrobial compounds and siderophores, enable strong and consistent suppression of wilt, damping-off, and early blight pathogens [113,114]. The diversity in VOC profiles and their antifungal activities varied across bacterial strains (Table 1), underscoring the potential of endophytic bacteria as natural biocontrol agents in managing plant diseases.

#### 5.1.3. Enzyme Production

The production of enzymes by antagonistic endophytes and epiphytes plays a pivotal role in the biocontrol of pathogens affecting tomato crops. Enzymes such as chitinases, proteases, and glucanases help degrade the cell walls of pathogens, effectively inhibiting their growth and enhancing the plant’s natural defense mechanisms [115,116].

Chitinases are among the most extensively studied enzymes in the context of biocontrol, primarily due to their ability to target chitin, a key component of fungal cell walls. Endophytic bacteria such as *Bacillus licheniformis*, have been shown to produce chitinase, which not only degrades the cell walls of pathogenic fungi but also induces systemic resistance in the host plant [117]. Several *Bacillus* strains, including *Bacillus megaterium* MB3, *B. subtilis*, and *B. amyloliquefaciens*, have demonstrated the ability to produce chitinase, β-1,3-glucanase, and proteases, particularly when exposed to the cell walls of *R. solani* as a carbon source. Molecular evidence, such as amplification of the chitinase (*chiA*) and β-1,3-glucanase genes, supports their antibiosis potential. In greenhouse trials, root treatment with these *Bacillus* strains enhanced the accumulation of defense-related enzymes (chitinase, glucanase, peroxidase, polyphenol oxidase, and phenylalanine ammonia-lyase) and total phenolic content in tomato leaves, providing protection against both fungal pathogens and insect pests [118]. Another study found that bacterial isolates (*Enterobacter cloacae* and *P. fluorescens*) produced significant amounts of hydrolytic enzymes such as protease, cellulase, chitinase, and lipase, which play a crucial role in degrading pathogen cell walls and inhibiting growth. *Enterobacter cloacae* and *P. fluorescens* showed notable inhibition against *Phytophthora capsica* [119]. These findings highlight the potential of these bacterial strains as effective biocontrol agents, reducing the impact of fungal pathogens like *Phytophthora capsici* through enzyme production and enhancing sustainable field crop protection.

Antagonistic microorganisms play an important role in protecting plants by boosting their natural defenses through triggering a process called systemic resistance, which strengthens the plant’s ability to fight off diseases [120]. For instance, *B. amyloliquefaciens* has been shown to help tomato plants resist bacterial wilt by increasing the activity of key defense enzymes like β-1,3-glucanase, peroxidase, and polyphenol oxidase [121] (Table 1). This ability to enhance a plant’s immune system shows how valuable these microorganisms are for sustainable and effective disease control.

#### 5.1.4. Iron Competition and Siderophore

Competition for nutrients and space in the rhizosphere is a key mechanism by which antagonistic microorganisms suppress plant pathogens. Rhizobacteria such as *Bacillus* spp. contribute to this process by producing siderophores-iron-chelating secondary metabolites synthesized under iron-limited conditions, which sequester ferric iron (Fe^3+^) and reduce its availability to pathogenic fungi. In tomato systems, several biocontrol agents produce siderophores to inhibit pathogens. Epiphytic *Pseudomonas* spp. and *Bacillus* spp. produce pyoverdine, pyochelin, and bacillibactin, respectively, while endophytic *B. bassiana* generates ferricrocin and fusarinine C, and *Streptomyces* spp. produce hydroxamate-, catecholate-, and carboxylate-type siderophores. These compounds restrict iron access to pathogens such as *F. oxysporum*, *B. cinerea*, and *Alternaria* spp., thereby enhancing plant protection [122,123,124]. Specifically, *P. fluorescens* not only limits iron for competing microbes, but also promotes rhizosphere colonization and can trigger induced systemic resistance in tomato. *Metschnikowia pulcherrima* produces pulcherrimin, further reinforcing iron limitation and competitive exclusion of pathogens [125]. Together, these strategies demonstrate that effective siderophore production suppresses pathogen growth, supports microbial persistence, and complements other antagonistic mechanisms (Table 1), establishing iron competition as a central factor in robust tomato disease management.

**Table 1:** Mechanisms of antagonism employed by microbial endophytes and epiphytes against plant pathogens.

Type	Microorganism	Target Pathogen(s)	Disease(s) Controlled	Volatile Organic Compounds	Antimicrobial Compounds	Lytic/Other Enzymes	Siderophores/Iron Competition	ISR/Other Mechanisms	Effectiveness	References
Epiphyte	*B. subtilis strains*	*B. cinerea*, *Alternaria*, *R. solani*, *Xanthomonas*	Gray mold, early blight, etc.	Acetoin, 2,3-butanediol	Surfactin, iturin, fengycin	Proteases, cellulases, glucanases	Bacillibactin	ISR; competition; biofilm	40–80% reduction	[107]
	*B. amyloliquefaciens*	*F. oxysporum*, *Botrytis*, *Alternaria*, *Pectobacterium carotovorum*	Fusarium wilt, gray mold, early blight, soft rot	Acetoin, 2,3-butanediol, 3-hydroxybutan-2-1	Bacillomycin D, fengycin, C12-C15 surfactin, iturin, fenycin	Proteases, chitinases	Bacillibactin	ISR; root colonization	Strong control, yield boost	[108,109]
	*L. capsici* AZ78	*P. infestans*	Late blight	Mono- and dialkylated methoxypyrazines	Dihydromaltophilin	Chitinases, proteases	No specification	Leaf colonization	Intermediate temps best	[110,111]
	*Metschnikowia pulcherrima*	*Botrytis*, *Alternaria*	Gray mold, early blight (some)	Fruity esters, alcohols	Pulcherrimin, killer toxins	β-glucanase	Pulcherrimin (strong iron binding)	Competition	Good on fruit; variable on foliage	[126]
	*B. cereus* HRT7.7	*R. solanacearum*	Bacterial wilt	VOCs not specified	Zwittermicin A, bacteriocins	Chitinases, proteases	Bacillibactin (reported)	Competition	Strong in vitro inhibition	[56,127]
	*Enterobacter hormaechei*	*R. solanacearum*	Bacterial wilt	Alcohols, ketones	Bacteriocins	Proteases	Enterobactin-type	Biofilm; competition	Reduced pathogen load on surface	[56]
	*Serratia marcescens*	*Pythium cryptoirregulare*	Damping-off	Blend (strain dependent)	Prodigiosin, serratamolide	Chitinases, proteases	Enterobactin-type	Biofilm; competition	Greenhouse suppression variable	[128]
Endophyte	*T. harzianum*	*Fusarium* spp., *R. solani*, *Pythium* spp., *B. cinerea*	Wilt, gray mold, root rot, damping-off	6-pentyl-2H-pyran-2-one	Peptaibols, gliotoxin, polyketides	Chitinases, glucanases, proteases	Iron competition	Mycoparasitism; ISR	50–90% reduction	[10,112]
	*Pseudomonas gessardii*	*Alternaria* spp., *C. lunata*, *X. vesicatoria*	Early blight, leaf spots	Variable VOCs (no specifications)	Phenazines, pyoluteorin	Proteases, chitinases	Pyoverdine-type (species-dependent)	Competition; colonization	Potent in vitro and leaf assays	[95,129]
	*Streptomyces* spp.	*F. oxysporum*, *B. cinerea*, *R. solanacearum*	Wilt, gray mold, root rots	2-Methylisoborneol, BetaMyrcene and 1-ethenyl-4-methoxy-benzene	Actinomycin D, filipin, others	Chitinases, glucanases, proteases	Hydroxamate, catecholate, and carboxylate	ISR; competition	Strong in vitro; variable field results	[122,130]
	*Paecilomyces formosus*	*Fusarium* spp., *Rhizoctonia*, *Botrytis*, *Rhizopus*	Root rots, damping-off, gray mold	Not specified	Broad-spectrum antifungals	Chitinases (genus)	Not central	Competition	Strong in vitro inhibition	[78]
Epiphyte/ endophyte	*P. fluorescens*	*F. oxysporum*, *A. solani*	Wilt, damping-off, early blight	Acetophenone, dimethyl trisulfide, volatile blends	2,4-DAPG, pyoluteorin, phenazine-1-carboxylic acid, 2-hydroxyphenazine, and phenazine-1-carboxamide	Chitinases, lipases, proteases	Pyoverdine, pyochelin	ISR; rhizosphere competence	Strong, consistent suppression	[113,114]
	*B. bassiana*	*B. cinerea*, *A. alternate*	Gray mold, early blight	Strain-dependent	Beauvericin, bassianolide, oosporein	Chitinases, proteases	Ferricrocin, fusarinine C	Dual action; growth promotion	Multiple isolates strong	[123,131]

VOCs—Volatile organic compounds, DAPG—Glyceraldehyde-3-phosphate dehydrogenase, ISR—Induced Systemic Resistance.

## 6. Impact of Cultivation Systems on Microbial Effectiveness

The success of biological control strategies using endophytic and epiphytic microorganisms in tomato cultivation is tightly linked to the specific environmental and agronomic context in which they are applied. Cultivation systems, particularly soil-based and hydroponic setups, differ significantly in terms of microbial community dynamics, abiotic conditions, and external inputs (e.g., pesticides, fungicides and fertilizers). These differences can profoundly influence microbial viability, colonization, and biocontrol efficiency [132].

### Soil-Grown vs. Hydroponic Systems

Soil-based systems host a naturally diverse and complex microbiome, including both beneficial and pathogenic microorganisms. This complexity can provide ecological stability and suppressive soil effects, but it may also pose challenges for introduced biocontrol strains due to intense competition and microbial antagonism. Soil acts as both a reservoir and a filter, influencing microbial access to plant roots and aerial tissues [133]. Anzalone et al. [134] found that soil and soilless tomato cultivation systems support distinct microbial communities. Soil-grown tomatoes had higher bacterial and fungal diversity, including beneficial strains of *Pseudomonas*, *Bacillus*, and *Streptomyces*, which are known for plant growth promotion and biocontrol. However, soil systems also harbored fungal pathogens such as *Fusarium*, *Verticillium*, *Alternaria*, *Olpidium*, and *Thielaviopsis*. It should be noted that within these genera (*Pseudomonas*, *Bacillus*, and *Streptomyces*), some isolates can also be pathogenic, highlighting the importance of strain-specific effects. In contrast, soilless systems had lower microbial diversity and fewer pathogens. Mejia et al. found that although soil- and hydroponic-grown tomato roots share a core microbiome, soil-grown plants host a significantly higher microbial diversity, with 777 unique genera compared to 133 in hydroponic systems [135].

Soilless systems, such as hydroponic setups, offer a more controlled environment than soil-based cultivation by using nutrient solutions and inert substrates like perlite or rock wool. While these systems generally exhibit reduced microbial diversity, microbial communities can still be established through water, seeds, air, plant roots, or handling. To restore beneficial functions such as pathogen suppression or promotion of plant growth, artificial inoculation with beneficial microorganisms are often employed [136]. Studies have shown that the reduced microbial competition in hydroponics can enhance colonization efficiency of applied microorganisms [132,137]. Kuryntseva et al. [137] compared the formation of endophytic bacterial communities inside plants grown in soil versus hydroponic systems. Their study showed that soil-grown plants host a more diverse and complex endophytic microbiome, while hydroponically grown plants support a more selective but stable community of endophytes. This research highlights how different cultivation methods influence endophytic colonization pathways and suggests that understanding these dynamics can improve the management of beneficial plant microbiomes in various production systems.

The cultivation system, whether soil-based or hydroponic, significantly influences the biocontrol efficacy and secondary metabolite production of antagonistic microorganisms. Ramudingana et al. [12] reported that endophytic fungi isolated from soil-grown tomatoes, including *P. africana*, and *C. micaceus*, exhibited stronger antagonistic activity against pathogens such as *R. stolonifera*, *F. solani*, *G. candidum*, *A. alternata*, *R. solani*, and *F. oxysporum*. In contrast, isolates from hydroponically grown tomatoes showed reduced antagonistic potential. A metagenomic investigation tracked the root microbiome of tomato seedlings transplanted from soilless substrates into soil. It revealed a dynamic shift: after transplantation, root microbial communities mirrored soil microbiota, with enhanced capacities for antibiotic biosynthesis pathways (e.g., macrolides, flavonoids), suggesting stronger biocontrol potential in soil-grown plants, even if initially planted in hydroponic-like substrates that primarily promote plant growth [138].

Emerging evidence suggests that metabolic outputs of the tomato plant, including carotenoids such as lycopene and β-carotene in the fruit, as well as organic acids such as citric, malic, and lactic acid in root exudates, are modulated by the cultivation system and may, in turn, influence microbial colonization and activity. Elevated levels of lycopene in hydroponically grown tomato fruits may indirectly affect microbial recruitment by modulating plant defense responses or oxidative stress signaling pathways. In contrast, organic acids actively secreted through root exudates can shape microbial community structure in the rhizosphere by altering pH and serving as carbon sources, thereby enriching acid-tolerant or metabolically compatible microorganisms, some of which exhibit antimicrobial activity or induce systemic resistance in the host plant [132].

In hydroponic systems, the overall microbial diversity is typically lower compared to soil, which can reduce competition and facilitate the targeted enrichment and functional screening of specific biocontrol strains such as *Bacillus*, *Pseudomonas*, *Trichoderma*, and *Streptomyces* spp., many of which have been successfully isolated from hydroponic tomato environments. In contrast, soil systems harbor a highly diverse and naturally competitive microbiome, providing a broader pool of potential antagonists but often posing challenges in isolating and establishing dominant strains due to niche overlap and strong microbial interactions [15,139]. Thus, understanding how cultivation practices shape the microbial community landscape is essential for optimizing biocontrol agent discovery, tailoring microbial consortia, and enhancing disease suppression in tomato production systems.

## 7. Metagenomic Approach for Antagonistic Microbial Diversity Assessment

**Figure 2:**
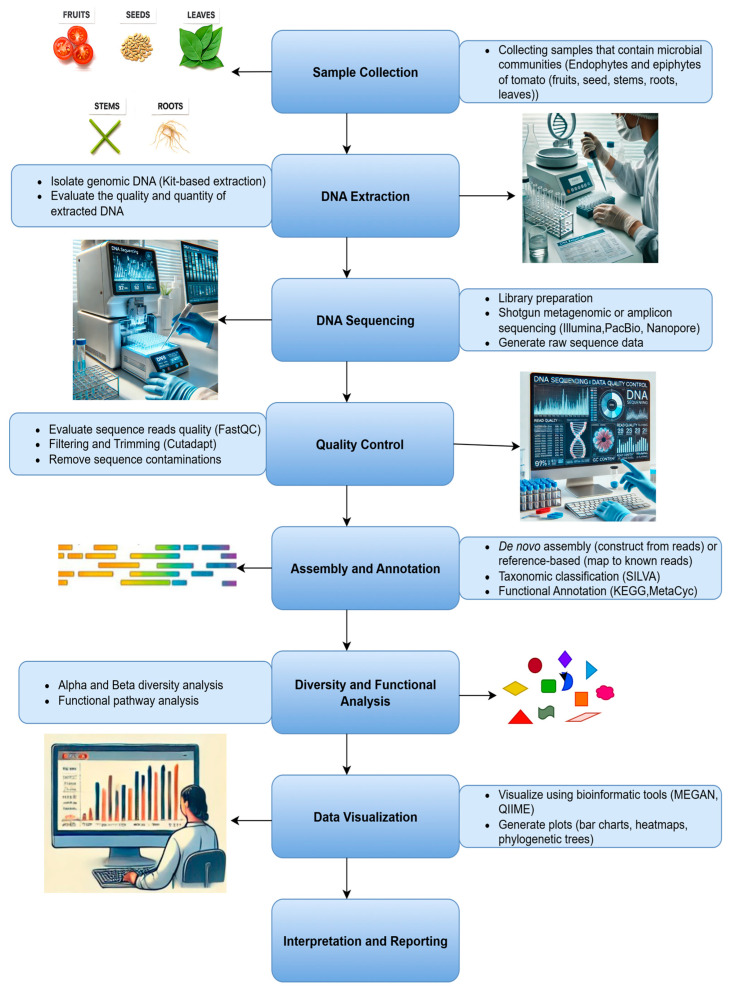
Detailed Workflow for the Metagenomic Analysis of Antagonistic Microorganisms. This figure outlines each key step—from sample collection and DNA extraction, through sequencing and bioinformatics analysis, to the final identification of microbial interactions—illustrating the comprehensive process involved.

Metagenomic approaches have become essential in unravelling the composition and functionality of microbial communities. Advancements in metagenomic techniques have revolutionized the study of antagonistic microbial diversity, particularly in agricultural biocontrol [140]. These tools enable the sequencing of collective genetic material from environmental samples, such as soil, plants, fruits, and rhizospheres, without the need for cultivating individual species. Metagenomics not only identifies microbial community structures but also reveals functional genes responsible for antimicrobial production, pathogen suppression, and plant growth promotion. Metagenomic analysis usually involves key steps such as sample collection, DNA extraction, library preparation, sequencing, data quality control, assembly and annotation, and downstream bioinformatic analysis to characterize the microbial community composition and functional potential [18] (Figure 2).

Most studies exploring microbial communities associated with antagonistic microorganisms, such as those found in tomatoes, use an amplicon-based metagenomic approach (e.g., 16S rRNA and ITS sequencing) to assess bacterial and fungal diversity. This technique effectively reveals taxonomic compositions and identifies prevalent genera. For instance, studies analyzing microbial populations on greenhouse-grown tomatoes using 16S rRNA sequencing identified *Bacillus*, *Pseudomonas*, *Citrobacter*, and *Enterobacter* as dominant genera [85]. However, while amplicon sequencing provides taxonomic insights, it cannot directly identify functional genes, such as those involved in the production of antimicrobial or volatile organic compounds [141]. Shotgun metagenomics overcomes these limitations by sequencing entire microbial genomes, offering direct insights into functional genes and metabolic pathways. For example, Nicotra et al. [142] employed high-quality genome sequencing (Oxford Nanopore long-read and Illumina short-read) to analyze bacterial strains from the core microbiome of tomato plants. Their findings revealed phyto-beneficial traits, including biofertilization, stress alleviation, and disease suppression, demonstrating the bacteria’s potential to combat diseases like Fusarium Crown Rot and bacterial spot infections.

Metagenomics employs high-throughput sequencing and advanced bioinformatics to provide a comprehensive view of microbial taxa and their potential roles [143]. For instance, Montagne et al. [144] used metagenomics to analyze soil and plant microbiomes, revealing diverse microbial consortia involved in nutrient cycling and pathogen suppression. The metagenomic study by Babalola et al. [145] revealed that the tomato rhizosphere is dominated by bacterial communities, particularly *Proteobacteria* and *Actinobacteria*, along with smaller proportions of *Acidobacteria*, *Bacteroidetes*, *Planctomycetes*, *Verrucomicrobia*, and *Firmicutes*. Fungi were also present but in low abundance (<1%), mainly from the *Ascomycota* and *Basidiomycota* phyla. These microbial groups are reported to play key roles in nutrient cycling, plant growth, and soil health.

The strength of the metagenomic approach lies not only in taxonomic profiling but also in the functional interpretation of microbial communities. Tools like Kraken v2.0.8 (beta) and MetaPhlAn v3.0.1 allow researchers to identify microbial genera with known antagonistic traits, while functional annotation against KEGG and MetaCyc v23.0 databases can reveal genes linked to secondary metabolite production, antibiotic biosynthesis, or stress resistance [146,147,148]. These insights are crucial when identifying microbial strains with biocontrol potential, as shown in studies where gene expression patterns in tomato-associated microbes revealed pathways involved in pathogen inhibition and plant growth promotion [20,143].

Moreover, visualization platforms such as Cytoscape v3.9.x and Phyloseq v1.38.0 help in interpreting these large datasets by highlighting microbial interactions, co-occurrence networks, and functional redundancies [146]. Such analyses help pinpoint key taxa that may act as keystone species in suppressing plant diseases. For example, Mamphogoro [17] isolated bacterial endophytes from tomatoes and used sequencing to identify strains with antagonistic properties against *Fusarium* species, offering promising leads for biocontrol applications.

### Functional Genes Involved in the Mechanisms of Tomato Biocontrol

Metagenomics identifies genes linked to critical biocontrol mechanisms (Table 2), such as antibiotic production, siderophore synthesis, and enzymatic activities that degrade pathogen cell walls. Furthermore, it sheds light on genes involved in plant growth promotion and the modulation of plant defense systems, offering integrated strategies for sustainable agriculture [140]. Mamphogoro et al. [149] sequenced the genome of *S. marcescens* SGT5.3, a plant growth-promoting bacterium isolated from a solanaceous fruit (*Capsicum annuum*). The 5.1 Mb genome contained 5019 genes, including those involved in nitrogen metabolism, indole-3-acetic acid (IAA) production, and siderophore biosynthesis.

Antagonistic microorganisms employ a combination of direct and indirect strategies to suppress tomato pathogens, integrating microbial activity with plant defense mechanisms. *P. fluorescens* contributes to biocontrol through the production of phenazines, pyoverdine (a siderophore), and chitinases. The functional genes *phz* and *pvdA* encode phenazines and pyoverdine, which enable the bacteria to sequester iron and inhibit fungal growth [95,150]. Paranthaman [151] demonstrated in planta that *P. fluorescens* secretes these metabolites to control soil-borne tomato pathogens, including *F. oxysporum*. *Trichoderma* spp. utilize a multi-layered biocontrol strategy. Genes encoding endochitinases (*chit42*) degrade chitin in fungal cell walls, directly limiting pathogen growth. Genes involved in nutrient competition (*GĴ1*) and siderophore production (harzianic acid) enhance *Trichoderma*’s ability to outcompete pathogens for iron and other essential resources in the rhizosphere. *Trichoderma* also produces polygalacturonases (*Thpg1*), which cleave plant-derived pectins to generate oligogalacturonides [152]. These oligogalacturonides act as damage-associated molecular patterns, activating plant innate immunity and systemic resistance pathways. By stimulating defense-related enzymes, such as phenylalanine ammonia-lyase and peroxidases, *Trichoderma* primes plants to resist subsequent fungal infections [153]. *T. harzianum* further contributes to pathogen suppression by producing chitinases and glucanases. The *chi1* gene encodes chitinase that degrades fungal cell walls, while *egl1* contributes to glucanase production, targeting fungal glucans [115,154]. Additionally, *Streptomyces* spp. produce a broad spectrum of bioactive compounds, including actinomycin D. Genes such as *act* and *str* encode the biosynthesis of actinomycin D and other antibiotics, demonstrating efficacy against tomato pathogens such as *Fusarium* and *R. solani* [93,98]. Ling [99] reported that *Streptomyces* spp. exhibit strong antifungal activity, partly due to these bioactive metabolites. Collectively, these functional genes enable antagonistic microorganisms to produce antimicrobial compounds and hydrolytic enzymes while simultaneously priming plant defenses, providing robust protection against a range of tomato pathogens.

**Table 2:** Genes involved in antagonistic activities of biocontrol microorganisms in tomatoes.

Organism	Gene	Function	Active Agent	References
*P. fluorescens*	*phlD*	Polyketide synthase	2,4-Diacetylphloroglucinol	[155]
*Trichoderma* spp.	*Thpg1*	Polygalactuonases; hydrolyzes plant pectins to generate elicitor molecules	Oligogalacturonides	[152]
*P. fluorescens*	*prnD*	Pyrrolnitrin biosynthesis	Pyrrolnitrin	[156]
*Pseudomonas* spp.	*hcnABC*	Hydrogen cyanide synthesis	Hydrogen cyanide	[157]
*B. subtilis*	*ituD*	Iturin biosynthesis	Iturin A	[158]
*B. subtilis*	*srfAA*	Surfactin biosynthesis	Surfactin	[159]
*Trichoderma* spp.	*chit42*; *GĴ1*	Degrades chitin; Mediates nutrient competition	Endohitinase	[152]
*B. amyloliquefaciens*	*bmyB*	Bacillomycin biosynthesis	Bacillomycin	[160]
*S. marcescens*	*chiA*	Chitinase biosynthesis	Chitinase	[161]
*T. harzianum*	*chi1*; *egl1*	Degrades chitin	Chitinase; Glucanase	[154]
*Pantoea agglomerans*	*nagB*	Chitin degradation	Chitinase	[162]
*Streptomyces murinus* NARZ	*PKS-I*, *PKS-II*, *NRPS*	Polyketide synthase, non-ribosomal peptide synthase	Various antifungal compounds (Pentamycin and actinomycin D)	[163]
*Streptomyces hydrogenans* and *Streptomyces* spp. NEAU-HV9	*Act*; *str*	Actinomycin D biosynthesis	Actinomycin D	[99,164]

## 8. Application and Commercialization of Endophytic and Epiphytic BCAs of Tomato

Endophytic and epiphytic BCAs provide a sustainable alternative to chemical pesticides in tomato disease management, but their transition from experimental success to widespread commercial use is constrained by several barriers. Laboratory and greenhouse trials consistently demonstrate their ability to suppress pathogens through antibiosis, volatile compounds, siderophores, hydrolytic enzymes, and induction of systemic resistance, yet field performance remains inconsistent [72]. For example, Reference [165] reported that *P. syringae* Cit7 and *Pseudomonas putida* B56 reduced foliar disease severity of bacterial spot in tomato, but did not consistently control fruit infection across sites, highlighting the ecological and environmental complexity of real-world conditions. A major challenge is ecological mismatch between the natural niche of BCAs and the chosen delivery method. Endophytes adapted to internal tissues often fail when applied as foliar sprays, while phyllosphere epiphytes show limited persistence in soil or seed environments. More promising outcomes are reported when delivery strategies align with microbial ecology: Akram [166] showed that seed coatings with endophytic bacteria significantly suppressed Fusarium wilt and improved seedling establishment, while Dania and Omidiora [167] demonstrated enhanced suppression of damping-off disease when microbial inoculants were combined with garlic extracts. These findings suggest that mimicking natural colonization routes and integrating plant-derived amendments can improve establishment and disease suppression in field conditions.

Formulation stability and shelf-life are additional bottlenecks. BCAs are formulated in different ways depending on the type of microorganism (spore-forming vs. non-sporulating) and the intended application (seed treatment, foliar spray, soil amendment). Formulation is essentially about stabilizing the microbes so they survive storage, transport, and environmental stresses while retaining biocontrol activity. Many non-sporulating organisms lose viability during production and storage, while spore-forming *Bacillus* strains have shown greater resilience under industrial fermentation and drying processes. Alginate encapsulation has been reported to extend the survival and field efficacy of endophytic BCAs under fluctuating environmental stress [168], while Calvo-Garrido [169] demonstrated that formulation type directly determined field success of *Bacillus ginsengihumi* against *B. cinerea*. These studies highlight that early integration of formulation science with strain selection is essential for commercial feasibility. Variability in field outcomes is also linked to climate, cultivar differences, and pathogen pressure. Multi-strain consortia offer a practical solution, as they combine complementary mechanisms and buffer against environmental variability. Microbial consortia consistently outperformed single isolates in suppressing tomato soil-borne pathogens across agroecological zones, while also improving yield stability [170].

The commercialization of BCAs for tomato disease management has advanced considerably over the last two decades, yet adoption is uneven and shaped by formulation stability, regulatory approval, and grower acceptance. Several products based on epiphytic and endophytic microorganisms have reached the market, demonstrating the potential of microbial inoculants as sustainable disease management tools. For instance, *B. subtilis* QST-713 (marketed as Serenade^®^, Bayer CropScience, Leverkusen, Germany) is one of the most widely commercialized BCAs. It is applied as a foliar spray against bacterial spot (*Xanthomonas* spp.) and fungal pathogens such as *B. cinerea* and *A. solani*. Serenade^®^’s success lies partly in its compatibility with copper-based fungicides, allowing it to be integrated into existing IPM programs rather than replacing chemical inputs entirely [171]. Similarly, *Trichoderma harzianum* T22 (sold as RootShield^®^—Granules, BioWorks, Inc., Victor, NY, USA) is used globally in greenhouse and open-field tomato production to protect against soil-borne pathogens like *P. ultimum* and *R. Solani*. Its commercialization was facilitated by its spore-forming resilience, ease of formulation, and consistent root colonization [172].

Other commercial successes illustrate the importance of multi-strain formulations and co-adjuvants. Products such as Eco-T^®^ (*Trichoderma atroviride*) and BioYield^®^ (a combination of *B. subtilis* and *Gliocladium virens*) gained traction because they combined complementary mechanisms, improving reliability under variable field conditions [173]. In hydroponic tomato systems, BCAs are often delivered alongside nutrient solutions or in combination with biostimulants, enhancing both disease suppression and plant vigor [136]. These cases demonstrate that successful commercialization typically requires integration with other agronomic tools, whether fertilizers, fungicides, or crop management practices.

## 9. Conclusions and Future Research

Endophytic and epiphytic biocontrol microorganisms present a sustainable and environmentally sound solution for managing plant diseases, particularly in intensive agricultural systems where over-reliance on chemical pesticides has contributed to environmental degradation, the emergence of resistant pathogens, and declining soil health. These beneficial microorganisms suppress pathogens and enhance plant health through multiple mechanisms, thereby reducing dependence on chemical inputs, slowing the development of resistance, and minimizing ecological harm. Recent advances in metagenomics and metabolomics have significantly improved understanding of microbial diversity and functionality, paving the way for more targeted and precise biocontrol strategies. Nevertheless, persistent challenges remain, including inconsistent effectiveness across cultivation systems, variable performance under field conditions, and limited knowledge of microbial interactions with plants and pathogens in diverse environments.

Future research should focus on the discovery of novel microbial strains with broad-spectrum activity, supported by genomic approaches to unravel their mechanisms of action, and validated through extensive multi-location field trials. To facilitate wider adoption, it is equally important to address commercialization challenges such as production costs, scalability, and regulatory hurdles. Furthermore, studying the compatibility of these microorganisms with existing chemical inputs can inform their integration into holistic disease management programs. Cutting-edge technologies such as synthetic biology hold great promise for enhancing metabolite production, improving stress tolerance, and engineering consortia with complementary traits. Innovations in formulation such as nano-encapsulation, polymeric carriers, and controlled-release systems can further improve microbial stability and field performance under fluctuating environmental conditions. In parallel, AI-driven predictive modeling and microbiome engineering are opening new avenues to design customized microbial consortia adapted to specific crops, soils, and climates. By integrating ecological insights with technological advances and farmer adoption, microbial biocontrol can be transformed from promising experimental tools into reliable, scalable technologies. Such progress will support the wider application of these agents, advancing sustainable agriculture while strengthening global food security.

## Data Availability

No new data were created or analyzed in this study. Data sharing is not applicable to this article.
